# Stable isotopes in the atmospheric marine boundary layer water vapour over the Atlantic Ocean, 2012–2015

**DOI:** 10.1038/sdata.2016.128

**Published:** 2017-01-17

**Authors:** Marion Benetti, Hans Christian Steen-Larsen, Gilles Reverdin, Árný Erla Sveinbjörnsdóttir, Giovanni Aloisi, Max B. Berkelhammer, Bernard Bourlès, Denis Bourras, Gaëlle de Coetlogon, Ann Cosgrove, Anne-Katrine Faber, Jacques Grelet, Steffen Bo Hansen, Rod Johnson, Hervé Legoff, Nicolas Martin, Andrew J. Peters, Trevor James Popp, Thierry Reynaud, Malte Winther

**Affiliations:** 1 Institute of Earth Sciences, University of Iceland, Reykjavik, Iceland; 2 LOCEAN, Sorbonne Universités, UPMC/CNRS/IRD/MNHN, Paris, France; 3 Centre for Ice and Climate, Niels Bohr Institute, University of Copenhagen, Denmark; 4 Department of Earth and Environmental Sciences, University of Illinois, Chicago, Illinois, USA; 5 LEGOS, UMR 5566 (University of Toulouse, CNES, CNRS, IRD, UPS), Institut de Recherche pour le Développement (IRD), CS 10070, 29280 Plouzané, France; 6 LATMOS—IPSL, Universite Pierre et Marie Curie, Paris, France; 7 Aix-Marseille Université, CNRS/INSU, IRD, Mediterranean Institute of Oceanography (MIO), UM 110, 13288 Marseille, France; 8 US191-Imago, Institut de Recherche pour le Développement (IRD), BP 70, 29280 Plouzané, France; 9 Bermuda Institute of Ocean Sciences, St George’s GE 01, Bermuda; 10 IFREMER, UMR 6523 LOPS (CNRS/IFREMER/IRD/UBO), CS 10070, 29280 Plouzané, France

**Keywords:** Atmospheric science, Ocean sciences

## Abstract

The water vapour isotopic composition (^1^H_2_^16^O, H_2_^18^O and ^1^H^2^H^16^O) of the Atlantic marine boundary layer has been measured from 5 research vessels between 2012 and 2015. Using laser spectroscopy analysers, measurements have been carried out continuously on samples collected 10–20 meter above sea level. All the datasets have been carefully calibrated against the international VSMOW-SLAP scale following the same protocol to build a homogeneous dataset covering the Atlantic Ocean between 4°S to 63°N. In addition, standard meteorological variables have been measured continuously, including sea surface temperatures using calibrated Thermo-Salinograph for most cruises. All calibrated observations are provided with 15-minute resolution. We also provide 6-hourly data to allow easier comparisons with simulations from the isotope-enabled Global Circulation Models. In addition, backwards trajectories from the HYSPLIT model are supplied every 6-hours for the position of our measurements.

## Background & Summary

The water vapour in the lower atmosphere is a key component of earth’s climate system. It is expected that the recent warming of the earth surface will strongly influence sea surface evaporation and precipitation patterns^1–3^. Hence in order to accurately project future changes in the atmospheric hydrological cycle, it is necessary to improve our understanding of the physical processes influencing the atmospheric hydrological cycle. Due to the molecular properties of water stable isotopes, isotopic fractionation occurs during phase transitions. This means that the isotopic composition of water vapour thus provides an integrated perspective on the hydrological history of an air mass. Comparisons between these observations and GCM simulations with isotope tracers can be used to test how accurate key processes such as evaporation, condensation and air mixing are captured by climate model simulations^4–6^. Evaporation is the starting point of the atmospheric hydrological cycle. The processes at play during evaporation are the main focus, which this dataset of marine boundary layer water vapor isotopes is applicable to.

Currently, the available datasets of isotope measurements of the atmospheric water vapor over the ocean are very limited^7–12^. Most of the measurements of atmospheric vapor isotopes were collected using traditional methods, involving collection of vapor samples by cryogenic trapping methods. Thanks to development of commercial water vapor isotope spectroscopy analyzers, it is now possible to provide continuous in-situ measurements. We provide here a combined new dataset of water vapor isotope measurements over the ocean with both large spatial and temporal resolution.

**Figure 1:**
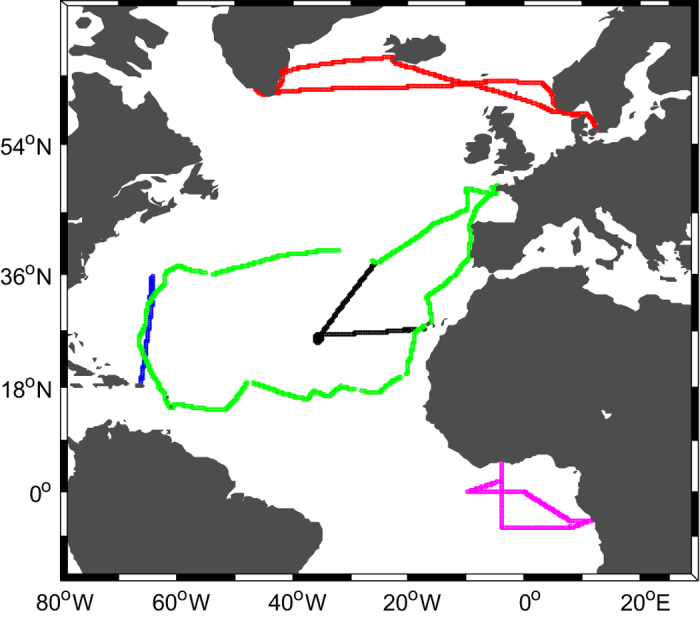
Map of continuous measurements provided during the 5 cruises between 2012 and 2015 over the Atlantic Ocean. In black: STRASSE, in magenta: PIRATA FR24, in green: RARA, in red: ACTIV, in blue: BERMUDA. The time periods are indicated in the Table 1.

This compilation of measurements covers a large part of the Atlantic Ocean, from 4° S to 63° N (see data distribution on Fig. 1). During five cruises, the atmospheric water vapor isotopic composition has been continuously measured with laser spectroscopy analyzers between 10 to 17 meters above the sea surface (Table 1). A weather station was installed on each vessel to continuously measure the standard meteorological variables (atmospheric sea level pressure, relative humidity, air temperature, wind speed and direction). In addition, during most cruises, near-surface ocean temperature measurements have been carried out at a depth between 3.5 and 1.5 m below the surface with a calibrated temperature probe (see Table 1).

The calibration of the raw Picarro data has been carefully performed against the international VSMOW-SLAP scale to obtain a homogeneous dataset for the five cruises, as described in the method section. All observations (Picarro and weather station measurements) have been averaged over 15 min and we also provide 6-hourly data for easy comparison with simulation outputs using isotope-enabled General Circulation Models. To facilitate interpretation of the dataset, we also provide relative humidity (RHS) normalized to sea surface temperature (SST) and backwards trajectories (120 h backwards) from the position of the measurements every 6 h using the HYSPLIT model^13^. Furthermore, we supply an averaged SST value, by using the OSTIA remote sensing product, over an area of 200 km by 200 km centred on the position of the ship.

We expect that this unique combined dataset for the Atlantic Ocean will contribute to:

1. Calibration of water stable isotopes observations from satellites^14,15^
2. Investigation of isotopic fractionation during oceanic evaporation and its relationship with atmospheric surface conditions^8,10,15–19^
3. Investigation of isotopic fractionation during condensation processes and characterization of convective and non-convective systems^9,12,15,20^
4. Evaluation and improvement of simulations from isotope-enabled General Circulation Models^4,15,21–23^
5. Improved paleoclimate reconstructions of humidity and surface temperature over the ocean^15,24,25^

## Methods

We describe here the protocol for producing the presented data in the following order:

1. Standard meteorological variables
2. Atmospheric water vapour isotope measurements
3. Calculated backwards trajectories

### Standard meteorological variables

Standard meteorological variables such as air temperature, relative humidity, wind speed, wind direction, and atmospheric sea level pressure were obtained from weather stations installed on the vessels. Furthermore, sea surface temperature (SST) has been continuously measured at a depth between 3.5 and 1.5 m below the surface with a calibrated temperature probe. The SST sensor placement was chosen to minimize the influence on the measurement from thermal heating caused by the research vessels. The heights and depths of sampling are presented in Table 1. The sensor names and their uncertainties are indicated in Table 2. The following subsections describe the measurement for each cruise.

#### The STRASSE cruise

A BATOS (French Met Office) weather station was installed at 18 m above the sea surface. The weather station was installed close to the centre of the ship, along the mast and above the bridge. The station consists of a contact protective housing (fan-aspired radiation shield) and was calibrated beforehand (for relative humidity, air temperature, wind, and pressure. During STRASSE, we also had at our disposal a dedicated instrumented mast near the bow (for turbulence measurements and also bulk measurements (Young Propeller 05106 anemometers and Vaisala HMP233 for humidity), which was used to check the consistency of the measurements. We found a discrepancy in the relative humidity, which we established with 10 measurements using a manual psychrometer. The applied correction is RH_Psychro_=0.9795×RH_Batos_−0.5263. The RH in the datafiles is the correct RH_Psychro_. The SST has been continuously measured by a calibrated temperature probe SBE35 at 3.50 m depth. The SST sensor has been calibrated before and after the cruise, with no indication of drift between successive calibrations larger than 0.01 °C. During the cruise, we have noticed occasional events of warming of less than 0.05 °C during stops at CTD cast stations (based on comparison with CTD cast data). We expect that the measurement of SST is accurate to within 0.05 °C.

#### The PIRATA FR24 cruise

A BATOS (French Met Office) weather station was installed at 12 m above the sea surface. The weather station was located portside above the bridge. The anemometer was installed above the weather station at a height of 18 m above the ocean surface. The BATOS weather station consists of contact protective housing (fan-aspired radiation shield) and has been calibrated beforehand (for relative humidity, air temperature, wind, and pressure. The SST has been continuously measured by a calibrated temperature probe SBE3S at 3.33 m depth. The SST sensors have been calibrated before and after the cruise, with no indication of drift between successive calibrations larger than 0.01 °C. We expect that the measurement of SST is accurate to within 0.05 °C.

#### The RARA cruise

A MetPak Pro weather station from Gill Instruments was installed at 4 m above the ocean surface. The weather station was located portside on the mizzenmast. The anemometer was installed above the weather station and the height of the wind measurement was 15 m. The Metpak Pro measurements are carried out in a fan-aspired radiation shield. The instrumentation consists of WindSonic ultrasonic wind speed and direction sensor, a highly accurate barometric pressure sensor, a Rotronic Hygroclip temperature/humidity probe, and a Pt100 probe for air temperature. During RARA AVIS cruise, the station was installed directly from the manufacturer and the conditions at sea on a sailing vessel precluded sophisticated calibration procedures. The near-surface seawater temperature has been continuously measured by a calibrated temperature probe SBE38 at 1.50 m depth. The temperature sensors have been calibrated before and after the cruise, with no indication of drift between successive calibrations larger than 0.01 °C. The measurement of SST is accurate to within 0.05 °C.

#### The ACTIV cruise

A DAVIS Vantage Pro 2 weather station was installed at 3 m above the sea surface. The weather station was located portside along the railing. The Davis measurements onboard ACTIV consists of a fan-aspirated radiation shield. The wind speed and direction could not be retrieved during the ACTIV cruise. We have instead used 6-hourly outputs from the ERA-interim reanalysis product^26^. No continuous SST measurements have been carried out during the ACTIV cruise. Instead we use estimates from the daily OSTIA product retrieved at the ship position^27^. However, no SST is reported close to the coast of Greenland for the period 15 July–5 September 2014, as they are not correctly estimated in the OSTIA products due to the size of the footprint. However, regular sampling of the sea surface temperature has been carried out throughout the campaign using a PT-100 thermistor submerged in ocean water immediately after sampling using a 10-litre bucket. These measurements are reported in the file SST_Activ_Bucket.txt. The good agreement (difference less than 0.1 °C) with the OSTIA estimations over the open ocean validated the measurement from the bucket, which can be used close to the coast of Greenland, where OSTIA estimations are not correct. In short, the measurement of surface water temperature is accurate to within 0.1 °C for the bucket measurement during the ACTIV cruise.

#### The BERMUDA cruise

A RM Young weather station was installed at 11 m above the sea surface, close to the centre of the ship, above the bridge. The anemometer was installed above the weather station and the height of the wind measurement was 15 m. The temperature and humidity sensors used for the BERMUDA system is obtained using a multi-plated radiation shield housing. The near-surface seawater temperature has been continuously measured by a calibrated temperature probe SBE3S (2 m depth). The measurement of surface water temperature is accurate to within 0.05 °C. We notice that the meteorological observations onboard the BERMUDA cruise are part of the permanent observational system on R/V Atlantic Explorer. The research vessel and operation is in compliance with the University National Oceanography Laboratory System.

#### Notes

The uncertainties on the meteorological data are given in Table 2 (factory values). It is possible that for some of the cruises, the accuracy is better, but we cannot guarantee it for all cruises. Notice that an observed constant difference between the Picarro measurements and the humidity measured by the weather stations for each cruise suggests that no significant change in the meteorological station measurements occured.

The meteorological station measurements can be influenced by the ship structure, both due to flow distortion, turbulent mixing, and heating/radiative influence from the ship itself. This is expected to contribute to the errors at least as much as the instrumental errors. Nevertheless, the comparison during the STRASSE cruise between the BATOS measurements, a dedicated mast near the prow and measurements 2-m above the sea surface suggested that the profile (of humidity, air temperature and wind) was consistent with the expected height of the BATOS measurements. Meteorological observations onboard R/V Atlantic Explorer were carried out both in starboard and portside, and the two sets of observations were not significantly different. Because RARA and ACTIV measurements were done on much smaller sailing vessels, it is expected that flow distortion is much less. However, during RARA and ACTIV, the presence of the sails nearby would certainly have modified the relative wind measurements. Nevertheless, we don’t notice major absolute wind changes when the RARA sailing vessel changed direction. No wind measurements are reported from ACTIV.

The larger issue is how to compare the measured seawater temperature with surface skin temperature. Tests done with COARE-Met flux algorithms suggest that the difference between measured temperature and the skin temperature is usually within 0.3 °C for those cruises due to near surface stratification and skin radiative cooling effects. Notice that there are however a few mid-day situations with very large near-surface stratification, when difference between the temperatures measured from the TSG and the radiative infrared temperature sensor that was installed for the STRASSE cruise was larger (caused by very weak winds during mid-day and early afternoon).

We have also calculated the relative humidity with respect to the sea surface temperature (RHS_10m) following equation 1.

(1)RHS-10m =qairqsat(SST)

With q_air_ being the specific humidity at 10 m, estimated from the Picarro measurement assuming that profiles follow the logarithmic profiles expected from the Monin-Obukhov similitude theory in a constant stress layer, *q*_*sat*_(*SST*) the specific humidity at saturation for a temperature equal to SST. q_sat_ is calculated for sea water at salinity 35, thus 2% lower than for freshwater^28^.

As for the ACTIV cruise, we also provide OSTIA measurements for the 4 other cruises (Resolution 1/20°). Then, to estimate horizontal heterogeneity of SST, we provide an average SST value over a square of 200 km by 200 km centred on the ship position (from OSTIA daily SST products).

### Data collected by the Picarro laser spectrometer

#### Experimental setup

Cavity-Ring-Down-Spectroscopy analyzers from Picarro Inc. were installed on each research vessel to measure the stable isotopic composition of the atmospheric water vapor and the specific humidity. The instrument versions of the different analyzers are presented in Table 1, as well as the main characteristic of the experimental setup. All the sampling inlets are located between 10 and 17 m above the sea surface. A protective inlet was installed at the beginning of the tube on all setups to prevent rain from being sucked into the tube and affecting the vapor measurements. The water isotopic composition is given in per mil following the definition by McKinney *et al.*^29^ and Craig^30^ whereas the humidity is reported in ppmv.

*The STRASSE cruise*. For the STRASSE cruise, the Picarro water vapor isotope instrument type was L2130i. The air is sampled from the atmosphere using ~10 m perfluolkoxy (PFA) tubing (outer diameter 13 mm, inner diameter 9 mm) with a 6 l/min airflow. The PFA tube was permanently heated to 40 °C or above to avoid condensation. The air has been sampled at ~17 m above the sea surface, just below the weather station BATOS.

*The PIRATA FR24 cruise*. For the PIRATA FR24 cruise, the Picarro water vapor isotope instrument type was L2130i. The air is sampled from the atmosphere using ~10 m perfluolkoxy (PFA) tubing (outer diameter 13 mm, inner diameter 9 mm) with a 6 l min^−1^ airflow. The PFA tube was permanently heated to 40 °C or above to avoid condensation. The air has been sampled at ~12 m above the sea surface, portside above the bridge, next to the weather station.

*The RARA cruise*. For the RARA cruise, the instrument type was L2130i. The air is sampled from the atmosphere using ~10 m perfluolkoxy (PFA) tubing (outer diameter 13 mm, inner diameter 9 mm) with a 6 l min^−1^ airflow. The air has been sampled at ~10 m above the sea surface from the fore mast. No heating was applied to the tube used on the RARA campaign. To check whether the lack of heating on the RARA cruise had any effect on the results we tested the setup at the beginning of the cruise by comparing the specific humidity measured by the Picarro analyzer with the measurements from the weather station, alternating periods of heating of the sampling tube with periods with no heating. In both situations we observe a consistent agreement with the humidity weather station measurements, indicating no condensation processes in the PFA tube. Moreover, at no time during the RARA cruise was the temperature in the tube below the dew point temperature.

*The ACTIV cruise*. For the ACTIV cruise, the instrument type was L1102i. The air is sampled using ~25 meter copper tubing (outer diameter ¼ inch) with a 6 l min^−1^ airflow. The copper tube was permanently heated to 40 °C or above to avoid condensation. The Picarro analyzer L1102 was situated in a PID temperature controlled enclosure. The air has been sampled at ~15 m above the sea surface, at the top of the mizzen mast.

*The BERMUDA cruise*. For the BERMUDA cruise, the instrument type was L2120i. The air is sampled using a ~20 meter stainless steel tube (outer diameter ¼ inch) with a 10 l/min airflow. Similar tube length (25 m) was used in the study of Steen-Larsen *et al.*^16^, in which the authors discussed the smoothing of the signal due do tubing length. No heating was applied to the tube used on the BERMUDA campaign, but an orifice was installed in the beginning of the tube to lower the pressure inside the tube and hence decrease the dew point temperature. The air has been sampled at ~11 m above the sea surface, along a mast located in front of the boat^31^.

*Notes*. It is not expected that sea spray affects the measurements as we did not find significant sea spray deposits on the tube and inlet at the end of the cruise. Moreover, conditions were never stormy enough to have major sea spray input during STRASSE, PIRATA FR24 and BERMUDA cruises. The good consistency between the humidity measured by the Picarro analyzer onboard and the humidity measured by the weather station suggests that no strong input of sea spray affected the measurement. Furthermore, if sea spray droplets were to be drawn into the inlet lines we would expect to see spikes in the humidity signal as the droplet evaporated. However, we cannot disregard the possibility that small sea spray droplets are drawn into the inlet and contribute to the water vapor measurements. If this were the case, we would expect that the measured isotopic composition to be more enriched than the ambient water vapor as the evaporation of sea spray would add vapor equivalent to the ocean isotopic composition.

Foam was placed underneath the instruments to dampen the vibrations and movements of the vessel on the Picarro instruments. No influence of the ship movements was observed during the STRASSE, PIRATA FR24, and BERMUDA cruise. However, since the RARA and ACTIV cruises took place on a sailing boat more significant vibration and movement was encountered. During those cruises, we observed that the cavity pressure fluctuated more during conditions with stong winds/high waves. Although it is difficult to precisely evaluate the effect of these variations on the isotopic measurement, we did not notice specific problems (e.g. changes in s.d. during calibration) during these periods of higher-pressure fluctuations. We furthermore notice that the cavity pressure variations are within the deviations approved by the Picarro manufacturer.

Finally, no influence of ambient room temperature variations on the water vapor isotope observations was observed during any of the cruises. The PID temperature-controlled enclosure, which housed the Picarro analyzer (model L1102i) during the ACTIV cruise minimized the temperature fluctuations to within 1 K. During the other cruises, the analyzers used (L2130i and L2120i) are much less sensitive to the temperature fluctuations, and did therefore not need a temperature regulated enclosure under these conditions.

The temporal resolution of the Picarro measurements depends on the instrument used. For the L1102i analyser the temporal resolution is about 20 s, while for the L2120/2130 analyser the temporal resolution is about 1 to 3 s depending on the instrument. We average these measurements for 15 min to obtain the high-temporal resolution dataset.

#### Calibration of the raw data

For the calibration of the raw data, we follow the protocol elaborated by Steen-Larsen *et al.*^32^ i. The first step is to correct the raw measurements of the concentration effect. ii. The second step is to convert the measurements to the international VSMOW-SLAP scale by using at least 2 different references of known isotopic composition. iii. Then, the measurements must be corrected for the instrumental drift by regularly injecting a reference standard to the analyzer.

**Figure 2:**
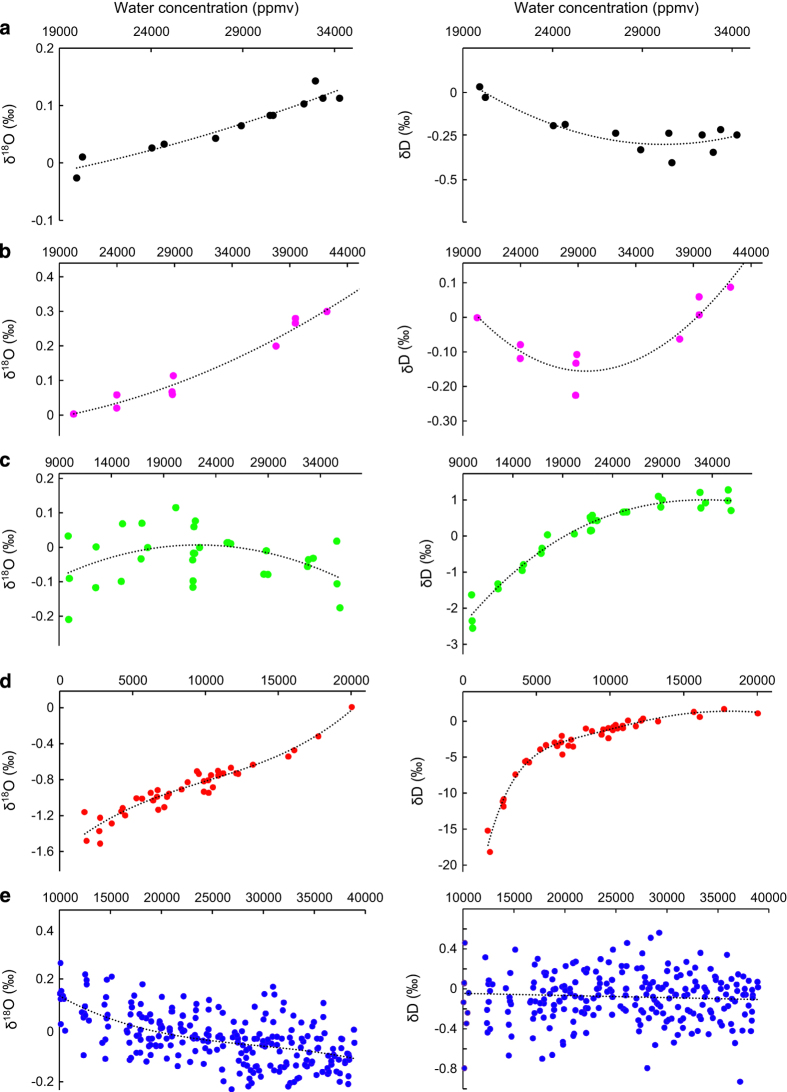
Evaluation of the concentration effect for the (a) STRASSE, (b) PIRATA FR24, (c) RARA, (d) ACTIV, and (e) BERMUDA cruise. Multiple evaluations have been carried out before, during, and after the cruises to validate the applied correction (see Table 6). The correction applied is shown by the black dotted regression curves (we chose 20 kppmv humidity level as a reference). For STRASSE, PIRATA and RARA, the tests have been done with a Picarro autosampler. The reproducibility of the method is 0.05‰ for δ^18^O and 0.40‰ for δD. For BERMUDA, each measurement corresponds to 4 min average at a given humidity. We estimate the precision for the 4 min averages to be better than 0.08 ‰ and 0.25 ‰ for δ^18^O and δD. For ACTIV, each measurement was carried out manually and corresponds to averaging over 10–15 min and the precision on the δ^18^O and δD measurements are about 0.04 ‰ and 0.20 ‰.

**Figure 3:**
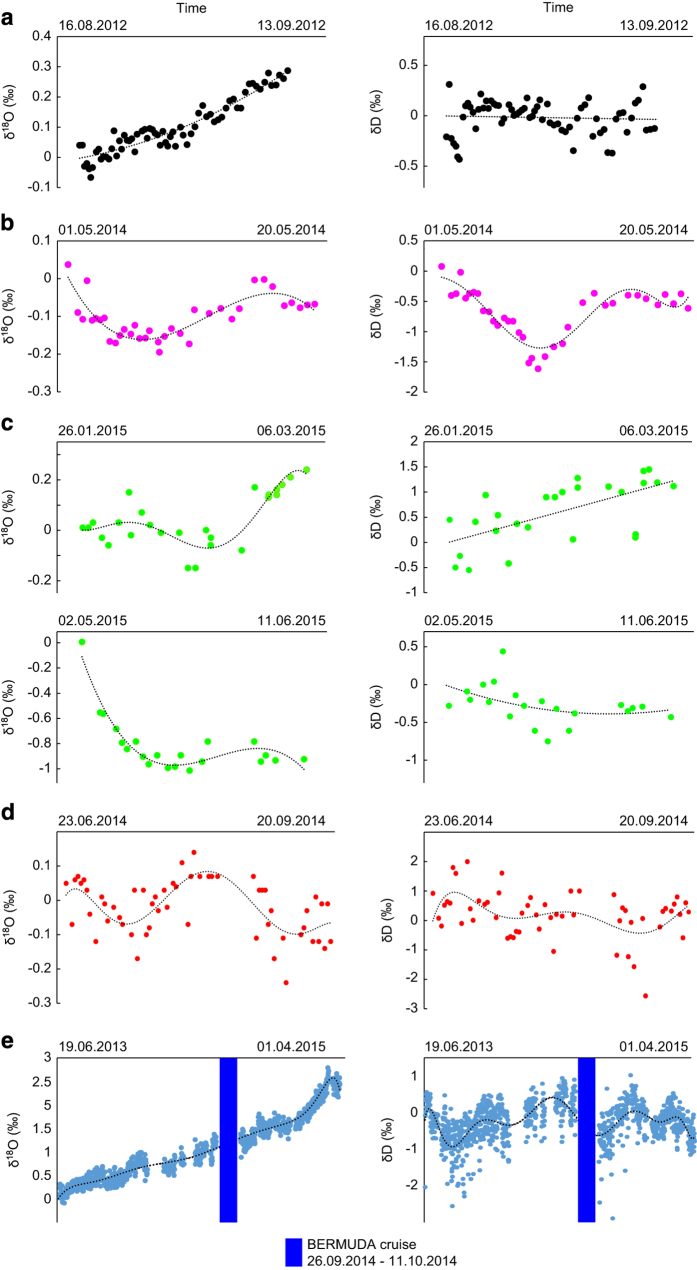
Instrumental drift of the Picarro laser spectroscopy analyzer for the individual instruments used during the 5 different cruises: (a) STRASSE, (b) PIRATA, (c) RARA (Leg 1 and Leg 2), (d) ACTIV and (e) BERMUDA. The x-axis corresponds to the full duration of the cruise, except for the Bermuda where no standard measurement has been done during the cruise (the period of the cruise is indicated by the blue box and the long term drift is shown over the period June 2013–April 2015). Linear interpolation is used across the period where no calibration was performed and lower accuracy of the Bermuda measurements is expected for this cruise compared to the 4 other ones. For the 4 other cruises, the regression curves used to correct the measurements are shown in black. For STRASSE and PIRATA, the tests have been done with a Picarro autosampler and the reproducibility of the method is 0.05‰ for δ^18^O and 0.40‰ for δD. For BERMUDA, ACTIV and RARA each measurement corresponds to at least 10 min average and we estimate the precision for the 10–15 min averages to be better than 0.10 ‰ and 0.25 ‰ for δ^18^O and δD.

- As described in Steen-Larsen *et al.*^32^ it is necessary to perform a humidity-isotope response calibration of the analyzer, and correct the raw measurements. The principle is to introduce a reference with a constant isotopic composition into the instrument at different humidity levels. Multiple humidity-isotope response calibrations were carried out before, during, and/or after each cruise. To correct the raw data from the concentration effect, we chose 20,000 ppmv humidity level as a reference similar to other studies^18,19,32,33^. The results of the humidity-isotope calibrations are shown in Fig. 2.
- Then, we reference the measurements against the International Atomic Energy Agency (IAEA) VSMOW-SLAP scale. We used at least 3 internal references bracketing the range of values encountered during the cruises. The values of the references used are reported in Table 3. The internal standards used to calibrate the water vapour isotope data have a typical uncertainty of (1 σ) of +/−0.03‰ for δ^18^O and 0.5‰ for δD. At least 2 calibration curves were established before or during each cruise to confirm the calibration in the VSMOW-SLAP scale. During each cruise, the slope of calibration curves did not evolve significantly during the period of the cruise.
- Finally, we regularly measured a reference standard to correct the instrumental drift through time. The evolution of the instrumental drift for each cruise is presented in Fig. 3 and the main characteristics of the protocol are described in Table 4.

The Picarro un-calibrated humidity measurements (in ppmv) have been calibrated against the specific humidity measured by the local weather stations on each of the cruises (see Table 2 for overview on instrument and uncertainty). For the five cruises, very high (R>0.95) correlation is obtained between the calibration curve and the measurements (the correction formula are reported in the Table 5). We report the calibrated Picarro humidity data in the data files supplied (in g kg^−1^ dry air).

The methods used for the calibration of the raw data are described for each cruise in Table 4 (ii.,iii.) and and in the following subsections.

*The STRASSE cruise*. We used a Picarro autosampler and vaporizer with compressed dry air (50 ppmv). The humidity-isotope response calibrations were done in the middle and at the end of the cruise. No shift in the humidity-isotope calibration was observed during the cruise. The drift of the instrument was measured by injecting a liquid reference every 6 h during the first 2 weeks. Later on we reduced the frequency of the reference injections, as the system proved to be very stable.

*The PIRATA cruise*. We used a Picarro autosampler and vaporizer with compressed dry air (50 ppmv). The humidity-isotope response calibrations were done in the middle and at the end of the cruise. No shift in the humidity-isotope calibration was observed during the cruise. To evaluate the instrumental drift, the measurements of the reference standard were carried out every 12-hour.

*The RARA cruise*. We used a custom-made calibration system described previously by Steen-Larsen *et al.* and Gkinis *et al.*^16,34^. We used ambient air, which was dried using Drierite (less than 200 ppmv). For this cruise, we did 3 humidity-isotope response calibrations carried out after the cruise when the instrument was back in the LOCEAN laboratory in Paris. To evaluate the instrumental drift, the measurements of the reference standard were carried out every 24–48 h.

*The ACTIV cruise*. We used a custom-made calibration system described previously by Steen-Larsen *et al.* and Gkinis *et al.*^16,34^. We used ambient air, which was dried using Drierite (less than 200 ppmv). The humidity-isotope calibration for the instrument used during the ACTIV cruise was carried out five times during the cruise and the combined dataset was used to correct the complete dataset. No shift in the humidity-isotope calibration was observed during the cruise. To evaluate the instrumental drift, the measurements of the reference standard were carried out every 24 h.

*The BERMUDA cruise*. We used a custom-made calibration system described previously by Steen-Larsen *et al.*^16^. We used ambient air, which was dried using Drierite® (less than 200 ppmv). This cruise was calibrated using a humidity-isotope response calibration carried out a few days before the cruise. As documented in Steen-Larsen *et al.*^15^, this instrument has been stable over multiple years. This was also supported by a humidity-isotope calibration carried out several months after the cruise. Unfortunately for the Bermuda cruise our calibration system failed during the period at sea and we therefore rely on the stability and knowledge of the long-term variability before and after the cruise from June 2013 to August 2015 (Fig. 3e). Linear interpolation is used across the period where no calibration was performed and lower accuracy of the Bermuda measurements is anticipated.

*Notes*. Besides a single short-term event during the ACTIV cruise, the ambient humidity level is never below 4,000 ppmv for the five cruises and we assume that the influence of the residual water in the dry air (<200 ppmv) on the Picarro measurement is insignificant^35^. In the following, each step of the calibration is described.

A recent study comparing directly water vapor isotope observations from two independently calibrated spectroscopy analyzers from Picarro documented an uncertainty on 10-minute average data of 0.14‰, 0.85‰, and 1.1‰ for δ^18^O, δD, and d-excess respectively^16^. Except for the data collected during the BERMUDA cruise, we expect this uncertainty to be representative of the data presented here. For the data collected as part of the Bermuda cruise we expect the uncertainty to be larger due to issues with the calibration system. However, we still expect, based on the long-term stability of the analyzer used for the Bermuda cruise, the combined accuracy and precision for the d-excess to be better than 2‰. We note the risk of overfitting both the humidity-isotope response relationship and the instrumental drift if too few measurements are used compared to the measurement precision. It is therefore important to always assess if the fitted trend is significant or not.

### Calculated backwards trajectories

Backwards trajectories have been calculated using the HYSPLIT—Hybrid Single Particle Lagrangian Integrated Trajectory Model (HYSPLIT OCTOBER 2014 release version) from the Air Resources Laboratory, NOAA^13,36^. Meteorological fields for the backwards trajectories have been obtained from NCEP’s Global Data Assimilation System 1-degree latitude-longitude resolution. Trajectories have been calculated backwards for 120 h from the 6-hour resolution position of the vessels. Trajectories were started 100 m above sea level. Elevation (m), air pressure (mb), air temperature (K), rainfall rate (mm/hour), marine boundary layer mixing depth (m), relative humidity (%), humidity mixing ratio (g/kg dry air), height of terrain (m), and solar insulation (W/m^2^) are given along the trajectory with a 1-hour resolution. Meteorological data along the back trajectory was obtained from the NCEP GDAS output.

## Data Records

All the files presented below are provided at http://cds-espri.ipsl.fr/isowvdataatlantic/ (Data Citation 1).

### Surface data

The surface data are recorded in ascii-format in the .txt-file and in matlab-format in the .mat-file. The variables are described in the Table 7. For each cruise (each vessel), two different files are created: one with a 15-minutes time scale resolution (average over 15 min) and another one with a 6-hours time scale resolution (average over 45 min centred on time stamp). The average is calculated for a given time stamp, only if 80% of the data are available. The naming convention is as follows: CRUISENAME_15_min.txt (.mat) or CRUISENAME_6_hour.txt (.mat). The columns of the .txt files are separated by a comma.

The additional file SST_Activ_Bucket.txt records the discrete SST measured using the 10-litre bucket (No interpolation on a equal time step is carried out). The variables are: Day Month Year Hour Min SST Latitude Longitude.

### Calculated backwards trajectories

The calculated backwards trajectories for each cruise (each vessel) are given in ascii-format in the .txt-file and in matlab-format in the .mat-file. The variables in the files are described in Table 8. The Matlab-format is the format of a structure array. The .txt-file is organized in blocks of 128 lines separated by 3 empty lines. The first 6 lines of each block give the starting time (year, month, day, hour UTC) and position (latitude and longitude). Line 7 of the block gives the header (see Table 8 for the description of the variables) of the output position and meteorological data along the individual trajectories. Each column is separated by a space.

## Technical Validation

**Figure 4:**
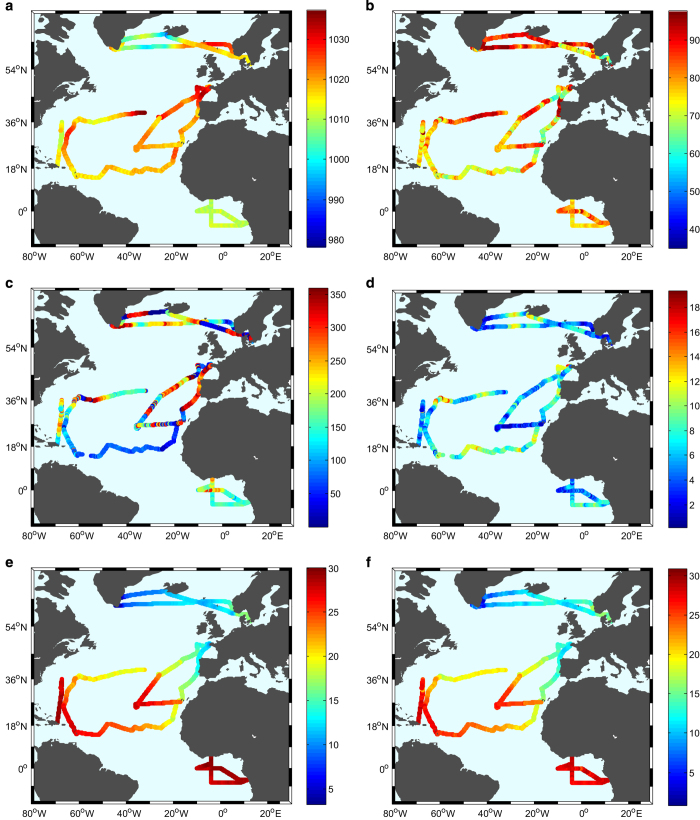
Spatial variability of (a) atmospheric sea level pressure in hPa, (b) relative humidity of the air in %, (c) wind direction (0° for northerly wind and 180° for southerly wind), (d) wind speed in m/s, (e) SST, and (f) air temperature (°C). For the ACTIV cruise, SST data are from OSTIA products and wind speed and direction are from the ERA-Interim reanalyses as discussed above.

**Figure 5:**
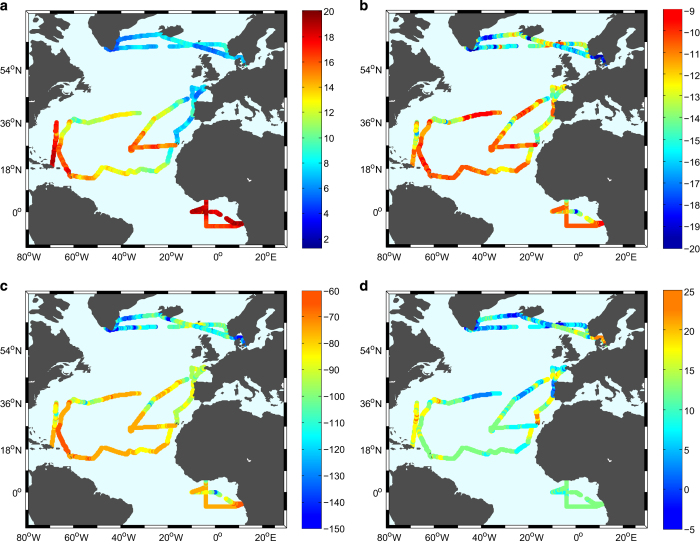
Spatial variability of (a) specific humidity in g.kg^−1^, (b) δ^18^O, (c) δD and (d) d-excess in per mill. Notice that the scale for the ACTIV cruise has been truncated due to the low values of δ^18^O and δD caused by katabatic winds in the Greenland Fjords (−35‰ for δ^18^O and −240‰ for δD) in order to better illustrate spatial variability. This means that all values below −20‰ in δ^18^O and −150‰ in dD are illustrated in the above figure with the same color coding. To allow better representation of the data of the BERMUDA campaign we have shifted its route slightly westward. The exact route of the BERMUDA campaign is shown in Figure 1.

- All the internal water standards used have been calibrated against the IAEA water standards (VSMOW, SLAP). Notice, however, that the internal water standards used to calibrate the isotopic Picarro measurement to the VSMOW-SLAP scale were not always the same for the different cruises.
- In the following, we present a brief description of the general weather conditions and discuss the general large-scale variability pattern of the surface conditions. The spatial variability of the main parameters discussed below is presented in Figs 4 and 5. The time series of the same parameters are presented in Supplementary Materials (Supplementary Figs S1).
- **STRASSE**
- This cruise took place in the eastern part of the subtropical North Atlantic gyre during the summer of 2012^18,19^. It can be separated in to three main parts: 1) From the Canary Islands to 26°N ; 35°W (survey area), 2) the survey area (26° N ; 35° W), 3) from 26° N ; 35° W to Ponta Delgada (Azores). The atmospheric sea level pressure increased from the Canary Islands to the study area, which is close to the Azores High pressure system (HP). During the survey, the atmospheric pressure fluctuated around 1,020 hPa, a mean value indicating the proximity of the HP (its core value was often close to 1,025 hPa). The surface winds usually came from the East or North-East, as expected for a trade wind regime. A small event of convection occurred around September 1st, with a 5 mb atmospheric pressure drop, and a small rain event as well as some isolated cumulonimbus were observed from the ship. Closer to the Azores Islands on the return leg, the atmospheric pressure decreased and rainfall events occurred with varying wind regimes. The largest air temperature (28 °C) was observed at the end of leg 1 and during the survey, but air temperature was more commonly near 26 °C with a slight diurnal cycle of 0.5 °C. Close to the Canary and Azores Islands, air temperature was lower (23 °C near the Canary Islands and 19 °C in Ponta Delgada). Specific humidity during the whole cruise ranged from 11 to 18 g kg^−1^ and RHS from 56 to 85%. The isotopic composition in the water vapour decreased during the two days of shallow convection and close to the Azores Islands.
- **PIRATA FR24**
- This cruise took place in the Gulf of Guinea from 9 April to 22 May 2014, with Picarro measurements during the 2nd leg from 1 to 20 May 2014. SST and Tair were generally higher than for the other cruises (27–30 °C) indicating warm surface conditions associated with the large solar fluxes close to the equator in late spring. A SST decrease around May 2 was associated with a weak equatorial upwelling event. The atmospheric sea level pressure did not change much during the cruise (from 1,008 to 1,014 hPa) as expected in the equatorial region. The mean value near 1,010 hPa is typical for this low-pressure region associated with the ascending branch of the Hadley cell. A period of deep convection occurred from May 3–7, close to the Intertropical Convergence Zone (ITCZ). At this time, the wind direction was variable, including some northerly winds at time. Another convection event was observed close to Abidjan (Ivory Coast) during the last day of the cruise. During these convection events, sudden air temperature decreases occurred. Except for these periods of convection, the region was dominated by a trade wind regime with winds coming mostly from the S-SE (135°–170°). During the whole cruise, specific humidity ranged between 15 and 20 g kg^−1^ and RHS between 60 and 83%. The most enriched water vapour isotope values were observed during periods dominated by the trade wind regime with limited atmospheric convection. The most depleted isotopic values were recorded during periods of strong convective activity when they dropped to −19‰ for δ^18^O and −140‰ for δD.
- **RARA**
- The RARA cruise is divided into two main legs.
- Leg 1 was from Brittany (Brest) to Martinique Island in the Antilles region during late winter 2015. Air temperature and SST progressively increased during the cruise from 10.5 to 26 °C and from 11.5 to 26.5 °C. This increase corresponds to the climatological latitudinal winter meridional gradient from Brittany to Cape Verde and zonal gradient from Cape Verde to Martinique. In the first part of the cruise, the ship crosses some of the cold waters carried by the winter upwelling off North West Africa. Closer to the Antilles, it encountered warmer and fresher waters of the western Atlantic. No deep atmospheric convection event was observed during this leg with specific humidity ranging from 5.6 to 15 g.kg^−1^and RHS from 51 to 100%. The driest air conditions were observed at the departure from Brest (clear sky), followed by a period of wet conditions in the Bay of Biscay associated with mist/fog over the sea surface. South of the Canary Islands, the wind direction was mostly NE veering to E after the Cape Verde Islands, which is characteristic of trade winds. SST/Tair measured in February in the South-Eastern part of the subtropical gyre was nearly 3 °C lower than during the summer STRASSE cruise, as expected from the seasonal cycle. The isotopic composition of the atmospheric water vapour ranged from −15 to −10‰ for δ^18^O and for −110 to −70‰ for δD.
- Leg 2 from Martinique to Brittany occurred in May and June 2015. The Picarro instrument was stopped during a week when the sailing boat was around the Azores Islands (by request of the ship’s master). The atmospheric sea level pressure increased from Martinique to the Azores Islands (except close to Bermuda, see discussion below), as the ship came closer to the center of the high-pressure system. At the Azores Islands, the highest pressure was 1,038 hPa. The temperature (air and sea surface) decreased during the crossing between the Antilles and towards Brittany, following the expected meridional gradient. Close to Bermuda, the ship encountered a strong convective event with atmospheric pressure dropping by 15 mb (on 12 May at 09 am, in the vicinity of 32.5°N and 64°W). The 15 min average wind speed reached 36 knots with sudden changes of directions. Estimated maximum wave height reached 8 m, and air temperature became much lower than SST (with the largest difference of 3.5 °C), suggesting unstable surface conditions.
- **ACTIV**
- The ACTIV cruise took place in the northern part of the North Atlantic between Scandinavia and Greenland throughout the summer of 2014. The cruise covers a large variability in atmospheric and oceanic conditions from the outer Baltic region to the fjords of south-east Greenland. This large variability also gave rise to a large span in d-excess values. Atmospheric specific humidity was most of the time lower than during the 4 other cruises and ranged from 1.3 to 10.3 g.kg^−1^. d-excess reached the highest value recorded by the 5 cruises (ranging from −6.3 to 25.7‰). The most depleted values of the isotopic composition, compared to the four other cruises has been observed during the voyage of ACTIV with values of −34.65‰ for δ^18^O and −255.49‰ for δD. During the cruise between Scandinavia and Greenland and for the return the large variability in water vapor isotope observations was governed by varying synoptic conditions. When the ship was near or in the fjords of south-east Greenland the varying water vapor isotope observations was governed by episodic katabatic winds coming off the Greenland Ice Sheet.
- **BERMUDA**
- The Bermuda-cruise took place in the autumn of 2014 between Bermuda and Puerto Rico and back as part of the Bermuda Atlantic Time-series Study validation cruise. Besides shorter periods of convective activity giving rise to heavy rain and rough sea near Bermuda at the beginning and the end of the cruise, the water vapor isotope composition (δ^18^O and δ D) is characterized by being very stable with values above −12‰ in δ^18^O. However, significant variations in the d-excess are observed throughout the entire cruise ranging between ~10 and 25 ‰. In agreement with the climatology, the SST latitudinal variability is less pronounced in October compared to this one measured in May in the same region with the RARA cruise (Fig. 4e).
- Isotopic measurement problems during the PIRATA FR24 cruise
- Due to high winds, the inlet cap protecting the tube from sucking in rain drops fell off during period from May 04th at 04:50 am to May 07th at 03:30 pm. This meant that during heavy rain events drops were sucked into the inlet line where it evaporated and influenced the water vapor isotope measurements. For this period, we have removed the data during and immediately after each rain event. We identified this issue from the differences between the weather station humidity measurements and the Picarro humidity measurements (the difference was always less than 0.7  g kg^−1^).

## Additional Information

**How to cite this article**: Benetti, M. *et al.* Stable isotopes in the atmospheric marine boundary layer water vapour over the Atlantic Ocean, 2012–2015. *Sci. Data* 4:160128 doi: 10.1038/sdata.2016.128 (2017).

**Publisher**’**s note**: Springer Nature remains neutral with regard to jurisdictional claims in published maps and institutional affiliations.

## Supplementary Material



Supplementary information

## Figures and Tables

**Table 1:** List of research cruises for this data set with a brief description of the equipment.

**Cruise**	**Ship name**	**Time period**	**Model Picarro (Height of inlet)**	**Tube (type and Length of tubing)**	**Weather station (Height of station)**	**Depth of water intake (m)**
STRASSE	R/V Thalassa	16.08–13.09 2012	L2130i (17)	PFA (10)	Batos (18)	3.50
PIRATA FR 24	R/V Suroit	01.05–20.05 2014	L2130i (12)	PFA (10)	Batos (12)	3.33
RARA	S/V Rara Avis	Leg1: 26.01–06.03 2015 Leg2: 02.05–11.06 2015	L2120i (10)	PFA (10)	MetPak Pro Gill (4)	1.50
ACTIV	S/V Activ	23.06–20.09 2014	L1102-i (15)	Copper (25)	Davis (3)	NN
BERMUDA	R/V Atlantic Explorer	26.09–11.10 2014	L2120i (11)	Stainless Steel (20)	RM Young (11)	2
The elevation above sea surface or depth below sea surface of the sampling is indicated in meters. During PIRATA FR24 (DOI 10.17600/14002100), RARA AVIS and BERMUDA, the anemometer was installed above the weather station: the height of the wind measurement was 18, 15 and 15 m, respectively. No continuous seawater measurements were carried out during the ACTIV cruise, but regular measurements have been done from manual measurements. No wind measurements have been done during the ACTIV cruise. The data acquired during the STRASSE cruise are published in Benetti *et al.*^18,19^.						

**Table 2:** Uncertainty on weather observations (factory values) and on the sea surface temperature measurements.

**Cruise**	**STRASSE**	**PIRATA FR24**	**RARA**	**ACTIV**	**BERMUDA**
*Air temperature*					
Sensor	Pt100	Pt100	Pt100	Davis Instruments	41382VF
Uncertainties	±0.15 K	±0.15 K	±0.1 K	±0.5 K	±0.15 K
					
*Relative humidity*					
Sensor	Vaisala HMP35DE	Vaisala HMP110	Hygromer IN-1	Davis Instruments	41382VF
Uncertainties	±3%	±3%	±0.8%	±3%	±3%
					
*Wind speed and direction*					
Sensor	Gill windsonic WS2	Vaisala WMT52	WindSonic ultrasonic	No measurement	51006 Wind Monitor—MA
Uncertainties	±0.3 m/s	±0.3 m/s	±2%	No measurement	±0.3 m/s
Uncertainties	±3 deg	±3 deg	±3 deg	No measurement	±3 deg
					
*Barometric pressure*					
Sensor	Vaisala PTB220	Vaisala PTB220	SCP1000	Davis Instruments	61302
Uncertainties	±0.15 hPa	±0.15 hPa	±0.5 hPa	±1.0 hPa	±0.15 hPa
					
*Sea surface temperature*					
Sensor	SBE35	SBE3S	SBE38	PT-100	SBE3S
Uncertainties	0.05	0.05	0.05	0.1	0.05
For the ACTIV cruise, regular measurements have been done from manual measurements, using a PT-100 thermistor submerged in ocean water immediately after sampling using a 10 litre bucket.					

**Table 3:** δ values of the references used to calibrate the Picarro measurements against the International Atomic Energy Agency (IAEA) VSMOW-SLAP scale.

**Cruise**	**References δ^18^O (‰)**	**References δD (‰)**
STRASSE	−0.56; −6.61; −15.81	−3.75; −44.32 ; −120.68
PIRATA FR24	−3.26 ; −6.61; −14.05	−21.32 ; −44.32; −100.96
RARA AVIS	−6.61; −14.05;−18.71; −19.74	−44.32 ; −100.96;−144.7; −146.53
ACTIV	+0.40; −0.02; −14.84; −33.44; −54.05	+2.8 ; −65.0 ; −111.1 ; −257.3 ; −424.1
BERMUDA	+0.40; − 33.56; −54.05	+2.8 ; −257.6 ; −424.1

**Table 4:** Main characteristics of the protocol of standard measurements.

**Cruise**	**Calibration system**	**Dry air source**	**Frequency of the drift measurement**	**Frequency of the VSMOW_SLAP calibration**	**Integrated time for measurement**
STRASSE	Picarro Autosampler	Compressed air (<50 ppmv)	~Every 6 or 12 h	~Every week (3)	8–20 injections
PIRATA	Picarro Autosampler	Compressed air (<50 ppmv)	~Every 12 h	Before and after cruise (3)	8–20 injections
RARA	Custom made calibration system	Drierite column (<200 ppmv)	~Every 24–48 h	4 times during the cruise (4)	10–15 min after a stable level
BERMUDA	Custom made calibration system	Drierite column (<200 ppmv)	Before and after cruise	Before and after cruise (3)	10 min after a stable level
ACTIV	Custom made calibration system	Drierite column (<200 ppmv)	~Every 24 h	~Every week (5)	10–15 min after a stable level
Standard measurements are necessary to evaluate the instrumental drift and to calibrate the raw data in the VSMOW-SLAP scale. The frequency of the drift measurement was adjusted along the cruise, and depends on the stability of the instrument during the cruise. The number in the parenthesis in column 5 indicates the number of standards used for the calibration. Their values are reported in Table 4.					

**Table 5:** Formula for converting un-calibrated humidity of the Picarro (Hum_pic) to calibrated humidity (Hum_cal).

**Cruise**	**Formula**
STRASSE	Hum_cal (in g/kg)=5.034 e-4 * Hum_pic (in ppmv)+9.850 e-2
PIRATA	Hum_cal (in g/kg)=4.942 e-4 * Hum_pic (in ppmv)+1.613 e-1
RARA AVIS	Hum_cal (in g/kg)=5.000 e-04 * Hum_pic (in ppmv)+3.139 e-1
ACTIV	Hum_cal (in ppmv)=−3.327e-5 * Hum_pic^2^+1.736 * Hum_pic (in ppmv)−4409
BERMUDA	Hum_cal (in ppmv)=0.927 * Hum_pic (in ppmv)+296

**Table 6:** Main characteristics of the protocol used to measure the humidity-response of the PICARRO instrument.

**Cruise**	**Calibration system**	**Dry air source**	**Number of tests**	**Integrated time for measurement**	**Humidity range for the tests (kppmv)**
STRASSE	Picarro Autosampler	Compressed air (<50 ppmv)	2	8 injections	20–35
PIRATA	Picarro Autosampler	Compressed air (<50 ppmv)	2	8 injections	20–43
RARA	Picarro Autosampler	Compressed air (<50 ppmv)	3	8 injections	9–37
BERMUDA	Custom made calibration system	Drierite column (<200 ppmv)	Before and after cruise following protocol of Steen-Larsen *et al.*^16^	4 min after a stable level	10–40
ACTIV	Custom made calibration system	Drierite column (<200 ppmv)	5	10–15 min after a stable level	1–20
The units of the humidity is kppmv. For each cruise, the humidity range for the test surrounds the humidity measured above the ocean during each cruise.					

**Table 7:** Name and description of the main variables included in the files.

**Variables**	**Unit**	**Description**
Time	Fraction of day since the 1st January of the year of the cruise	Date/time of TSG measurement in UTC
Year, Month, Day, Hour, Min	—	These variables indicates the date in UTC
Latitude	Decimal degree	Latitude of TSG measurement (negative values for South hemisphere)
Longitude	Decimal degree	Longitude of TSG measurement (negative values for west from Greenwich meridian)
Specific_humidity	g/kg	Specific humidity in g/kg measured by the PICARRO (calibration based on the weather station measurement)
d18O_VSMOW	‰ (VSMOW)	Isotopic composition in Oxygen 18 in the water vapour
dD_VSMOW	‰ (VSMOW)	Isotopic composition in Deuterium in the water vapour
XS_VSMOW	‰ (VSMOW)	Deuterium excess
SLP	hPa ou mbar	Atmospheric sea level pressure (SLP)
RH	%	Relative humidity at air temperature
Wind_Direction	°	(0° for northerly wind and 180° for southerly wind) (no measurement during ACTIV)
Wind_Speed	m/s	Wind speed (no measurement during ACTIV)
Wind_Direction_ERA	°	Wind direction from 6-hourly outputs from the ERA-interim reanalysis product (only for the ACTIV cruise)
Wind_Speed_ERA	m/s	Wind speed from 6-hourly outputs from the ERA-interim reanalysis product (only for the ACTIV cruise)
Air_temp	°C	Air temperature
SST _TSG	°C	Temperature measured by the sea water temperature probe (no values for ACTIV cruise*)
RHS_10m	%	Relative humidity normalized to SST _TSG (no values for ACTIV cruise†)
SST_OSTIA_Larger	°C	SST from OSTIA products (over a square of 200 km by 200 km centered on the ship position)
SST_OSTIA_Local	°C	Local SST from OSTIA SST daily products (Resolution 1/20°)
There are 2 files for each cruise (15 min, 6 h). The variable SST_TSG is not available for the Activ files. For the Activ cruise, the wind measurement (wind and direction) is from 6-hourly outputs from the ERA-interim reanalysis product^26^. The specific humidity in g/kg can be calculated in ppmv by dividing the mixing ratio with 621.9907 and multiply with 1e6.		

*SST_bucket_interp_greenland is a variable added for the ACTIV cruise. It contains the SST measured from the bucket (see section 2) interpolated linearly to the 15 min and 6 h time scale during the time when the vessel was close to Greenland coast, where the OSTIA fields are strongly biased. Due to large variations of the SST in the Greenlandic fjords due to mixing of glacier meltwater, the values for this part of the ACTIV cruise should be used with caution.

†For the ACTIV cruise we have added the variable RHS_10m _OSTIA, which represents the RHS, except around the Greenland coast, calculated using SST_OSTIA_Local and RHS_10m _bucket_greenland, which represents the RHS around the Greenland coast, calculated using the SST measured from the discretely sampled bucket-observations and given in the SST_bucket_interp_greenland file.

**Table 8:** Name and description of the variables included in the files containing the backwards calculated trajectories using the HYSPLIT model.

**Variables**	**Unit**	**Description**
Year, Month, Day, Hour		The time in UTC for a given position of a calculated air mass backwards trajectory
Backward_Hour	hours	Time since start of particle at position of observation
Latitude and Longitude		Calculated position of air mass particle along trajectory
Elevation	meters	Calculated elevation of air mass particle along trajectory
Air_Pressure	millibar (mb)	Air pressure at position and elevation of air mass particle along trajectory
Air_Temp	Kelvin (K)	Air temperature at position and elevation of air mass particle along trajectory
Rain_Fall	mm/hour	Rain fall rate at the position of the air mass particle along trajectory
Mixing_Depth	meters	Mixing depth of boundary layer at the position of the air mass particle along trajectory
RH_Air	%	Relative humidity of the air at the position and elevation of the air mass particle along the trajectory
Mixing_Ratio	g/kg of dry air	The humidity mixing ratio at the position and elevation of the air mass particle along the trajectory
Terrain_height	meters	Height of the terrain at the position of the air mass particle along the trajectory
Solar_Insulation	W/m^2^	Downward solar radiation flux at the position of the air mass along the trajectory
