# Supplementary material for: Stable isotopes in the atmospheric marine boundary layer water vapour over the Atlantic Ocean, 2012–2015
Source: Sci Data. 2017 Jan 17;4:160128. doi: 10.1038/sdata.2016.128 (PMC5240618; doi:10.1038/sdata.2016.128)
Supplement: Supplementary information [file sdata2016128-s2.pdf]

Supplementary materials

Title

Stable isotopes in the atmospheric marine boundary layer water vapour over the Atlantic Ocean, 2012-2015

Authors

Marion Benetti[12]*, Hans Christian Steen-Larsen[3]*, Gilles Reverdin[2], Árný Erla Sveinbjörnsdóttir[1], Giovanni Aloisi[2], Max B. Berkelhammer[4], Bernard Bourlès[5], Denis Bourras[6], Gaëlle de Coetlogon[6], Ann Cosgrove[4], Anne-Katrine Faber[3], Jacques Grelet[7], Steffen Bo Hansen[3], Rod Johnson[8], Hervé Legoff[2], Nicolas Martin[2], Andrew J. Peters[8], Trevor James Popp[3], Thierry Reynaud[9], Malte Winther[3]

*Both authors contributed equally to this publication

Affiliations

1. Institute of Earth Sciences, University of Iceland, Reykjavik, Iceland

2. LOCEAN, Sorbonne Universités, UPMC/CNRS/IRD/MNHN, Paris, France
3. Centre for Ice and Climate, Niels Bohr Institute, University of Copenhagen, Denmark
4. Department of Earth and Environmental Sciences, University of Illinois, Chicago, Illinois, USA

5. LEGOS, UMR 5566 (University of Toulouse, CNES, CNRS, IRD, UPS) Institut de Recherche pour le Développement (IRD), BP 70, 29280 Plouzané, France
6. LATMOS – IPSL, Universite Pierre et Marie Curie, Paris, France

7. US191-Imago, Institut de Recherche pour le Développement (IRD), BP 70, 29280 Plouzané, France
8. Bermuda Institute of Ocean Sciences, St. George's GE 01, Bermuda.
9. IFREMER, UMR 6523 LOPS (CNRS/IFREMER/IRD/UBO), CS 10070, 29280 Plouzané, France

Corresponding authors: Marion Benetti (marioncb@hi.is) and Hans Christian Steen-Larsen (hanschr@gfy.ku.dk)

Table of content

Page 2 : Figure S1

Page 3 : Figure S2

Page 4 : Figure S3

Page 5 : Figure S4

Page 6 : Figure S5

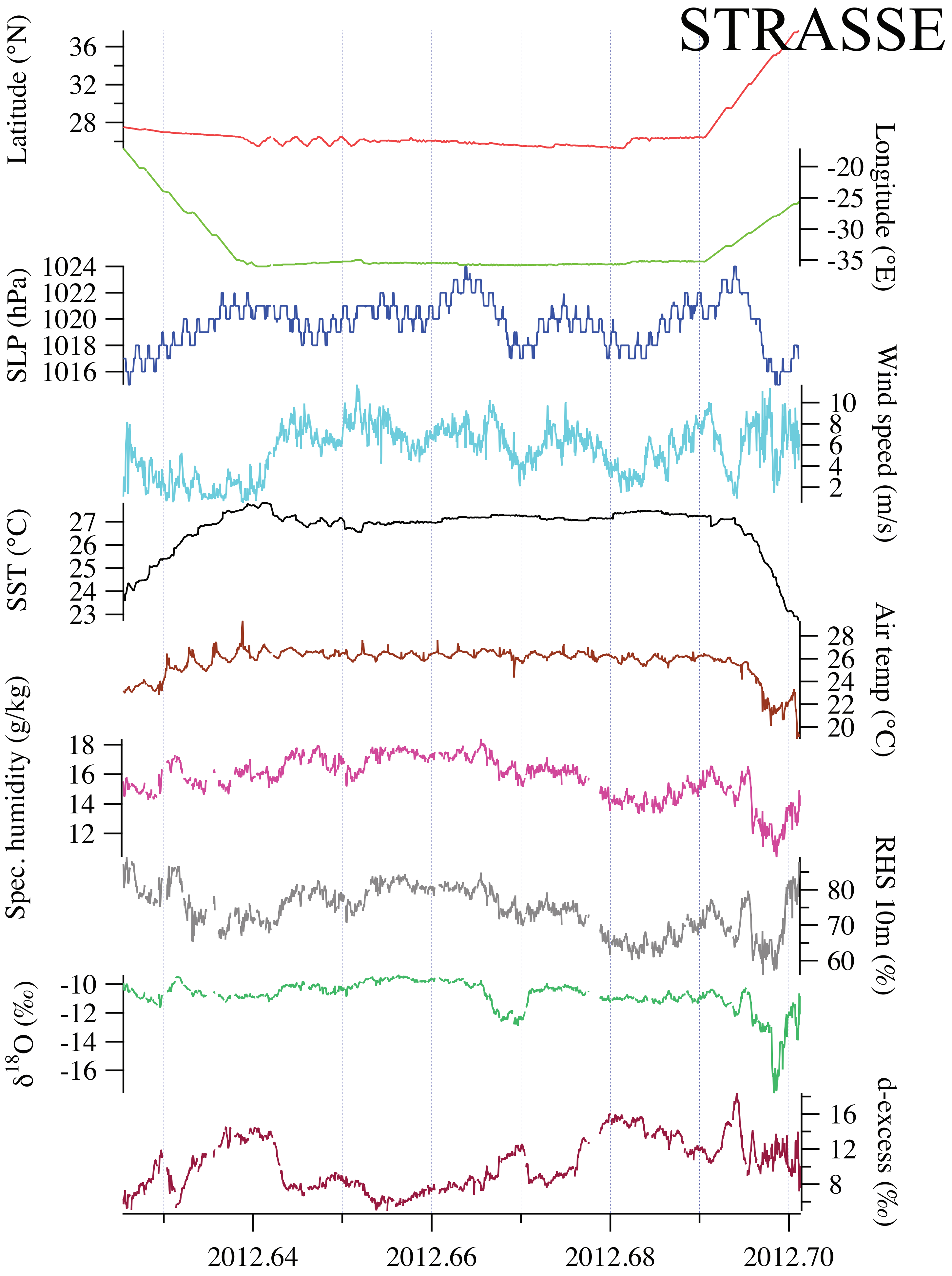

Figure S1 : Evolution versus time of the standard meteorogical variables during the STRASSE cruise. The variable units are the same as in Figure 4 and 5. The time is indicated in fraction of years.

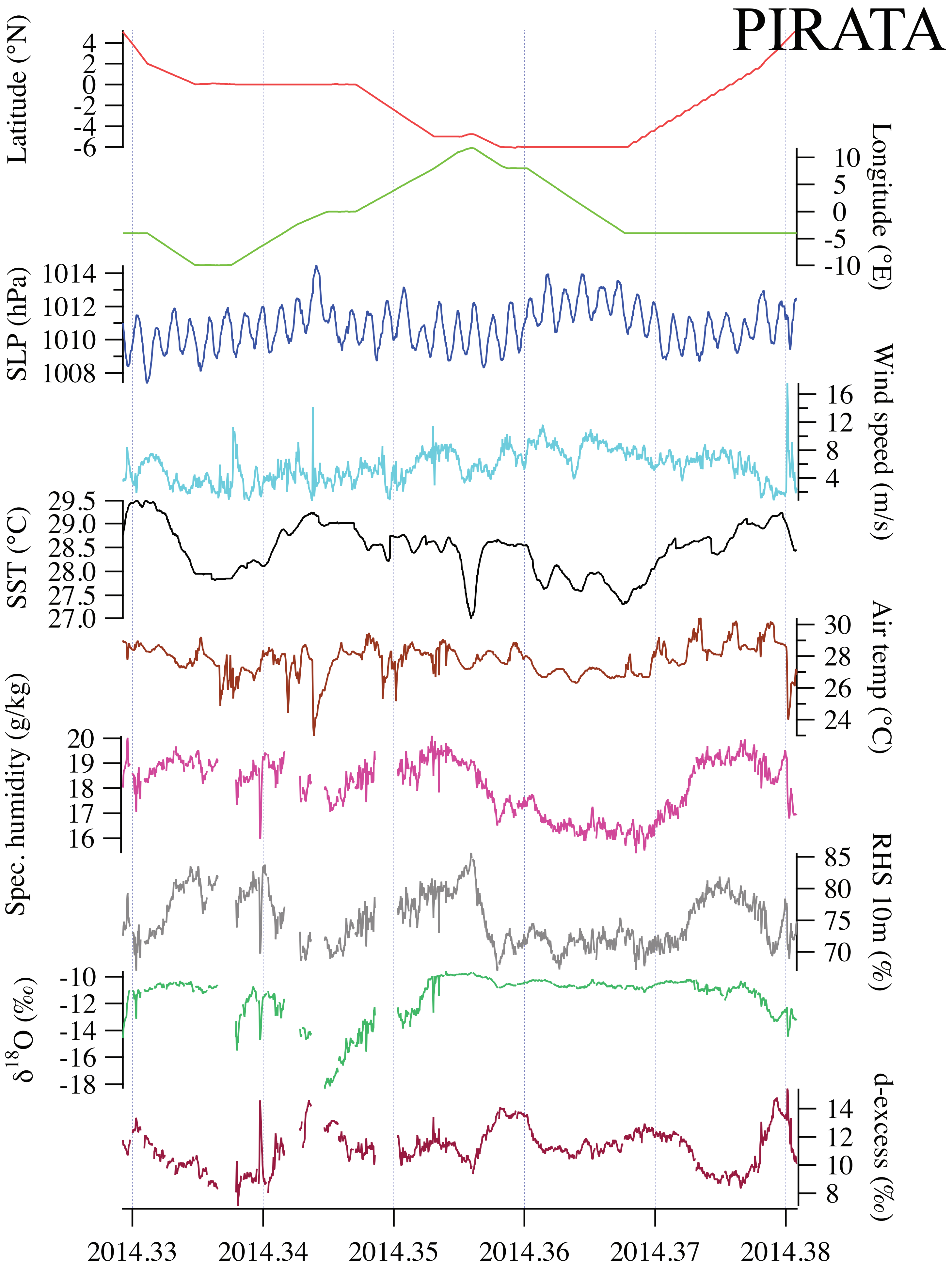

Figure S2 : Evolution versus time of the standard meteorogical variables during the PIRATA cruise. The variable units are the same as in Figure 4 and 5. The time is indicated in fraction of years.

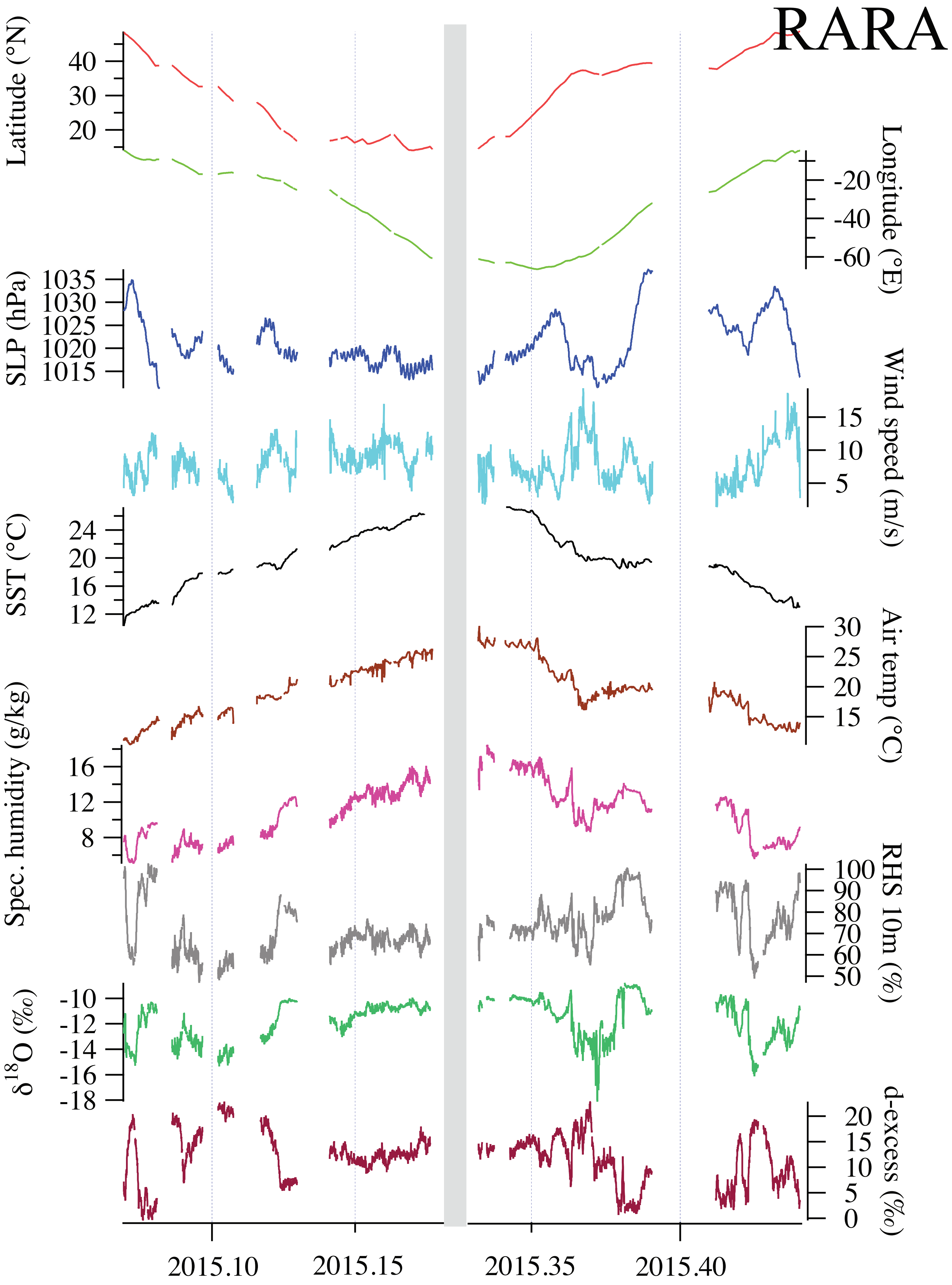

Figure S3 : Evolution versus time of the standard meteorogical variables during the RARA AVIS cruise. The variable units are the same as in Figure 4 and 5. The time is indicated in fraction of years.

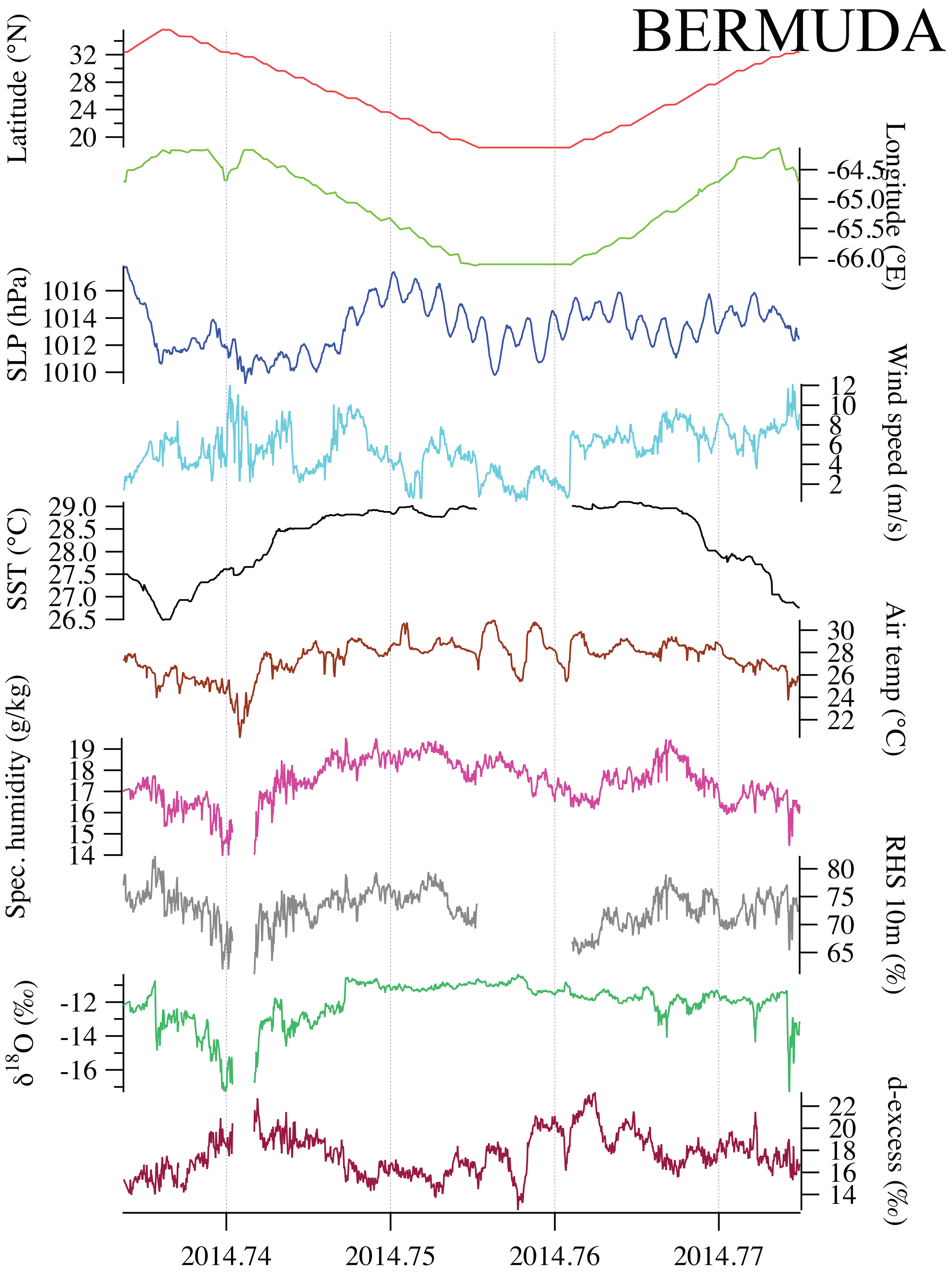

Figure S4 : Evolution versus time of the standard meteorogical variables during the BERMUDA cruise. The variable units are the same as in Figure 4 and 5. The time is indicated in fraction of years

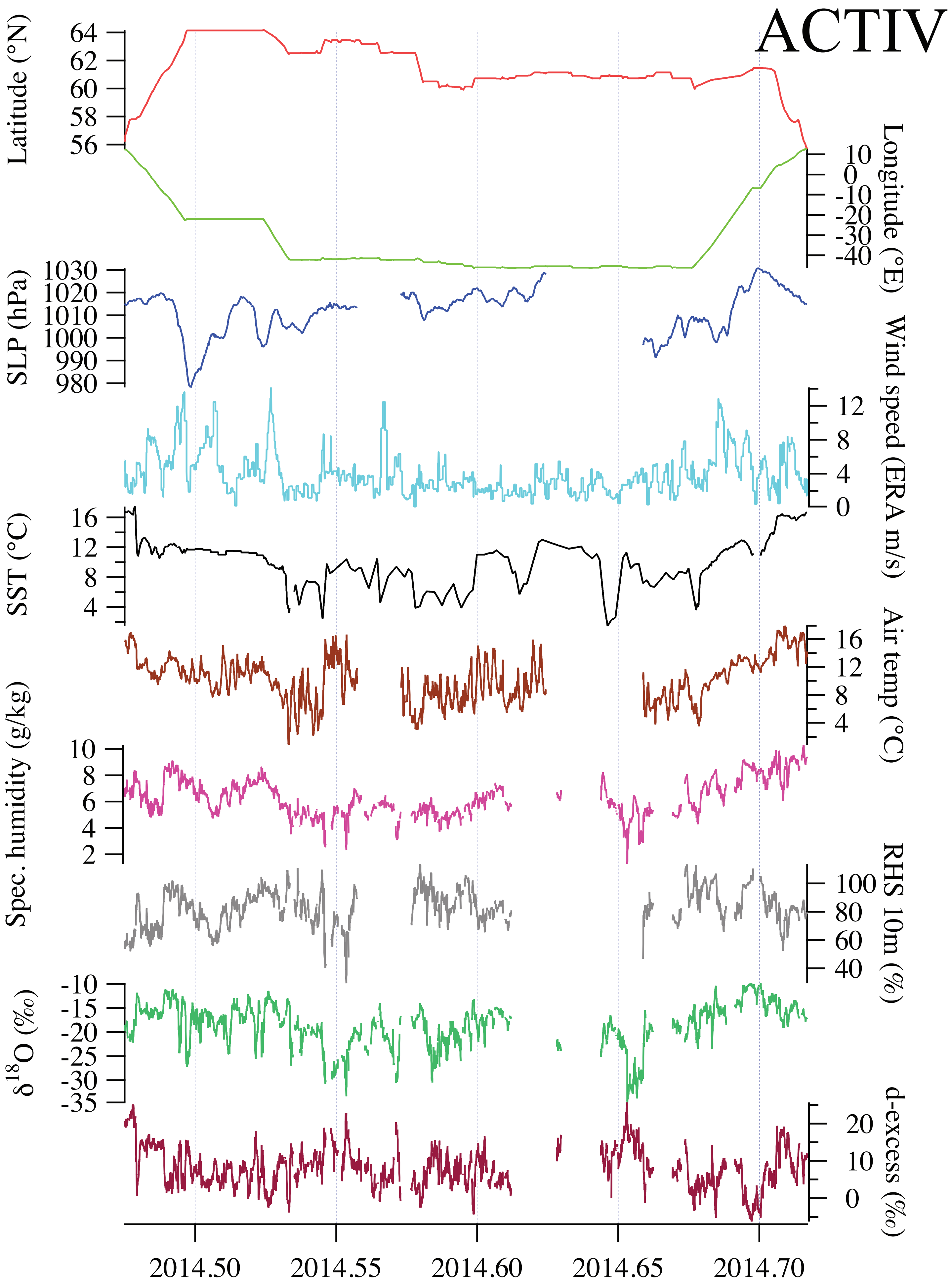

Figure S5 : Evolution versus time of the standard meteorogical variables during the ACTIV cruise. The variable units are the same as in Figure 4 and 5. The time is indicated in fraction of years. For the ACTIV cruise, SST data are from OSTIA products and wind speed are from the ERA-Interim reanalyses.
